# Computational and Statistical Analysis of Protein Mass Spectrometry Data

**DOI:** 10.1371/journal.pcbi.1002296

**Published:** 2012-01-26

**Authors:** William Stafford Noble, Michael J. MacCoss

**Affiliations:** 1 Department of Genome Sciences, University of Washington, Seattle, Washington, United States of America; 2 Department of Computer Science and Engineering, University of Washington, Seattle, Washington, United States of America; University of California San Diego, United States of America

## Abstract

High-throughput proteomics experiments involving tandem mass spectrometry produce large volumes of complex data that require sophisticated computational analyses. As such, the field offers many challenges for computational biologists. In this article, we briefly introduce some of the core computational and statistical problems in the field and then describe a variety of outstanding problems that readers of *PLoS Computational Biology* might be able to help solve.

This is an “Editors' Outlook” article for *PLoS Computational Biology*

## Introduction

DNA gets a lot of attention these days, in whole genome sequencing projects, genome-wide association studies, and experiments measuring transcription factor binding, chromatin accessibility, DNA methylation, and histone modification profiles. But proteins are the molecular workhorses of the cell, and proteomics—the systematic study of the complete set of proteins expressed in a given cell, tissue, or organism—is poised to become the next hot topic.

Just as next-generation sequencing tehcnology is driving the current genomics boom, so improvements in tandem mass spectrometry technology are leading to more comprehensive and precise proteomics assays. Like a short-read sequencing machine, a mass spectrometer runs 24 hours a day, producing a huge quantity of data. And like short-read sequencing data, mass spectral data sets exhibit complex dependencies and patterns of missing data. In both fields, the underlying technologies, along with the characteristics of the resulting data sets, change rapidly, requiring constant development of new analytical methods.

Strikingly, however, relatively few bioinformatics researchers work on methods for analyzing mass spectrometry data. *PLoS Computational Biology* published only two papers on the topic in 2010 [1], [2]. At the Intelligent Systems for Molecular Biology (ISMB) conference, none of the designated subject areas for submitted manuscripts is relevant to mass spectrometry analysis, and over the last three years, a total of four mass spectometry papers were published at ISMB, each appearing in the “Other bioinformatics applications” category. Meanwhile, the annual American Society for Mass Spectrometry conference draws more than 6,000 attendees, and the society boasts 7,000 current members.

Some of the forces preventing people from entering the mass spectrometry research arena are social. As a field, mass spectrometry is older than genomics, and as such, the norms around the sharing of mass spectrometry data are less open. In addition, intellectual property issues, such as the SEQUEST patents held by the University of Washington, may have discouraged some researchers from entering the field.

An equally important impediment, however, is the “energy barrier” associated with starting out in mass spectrometry. In the late 1990s, microarray analysis took off with surprising rapidity, in part because the data could be fairly accurately summarized in matrix format, and manipulating matrices is familiar to computer scientistics, electrical engineers, and physicists. This is not the case with mass spectrometry data. If you show someone with no relevant background knowledge a DNA sequence or a microarray image, explaining what they are looking at will take less time than explaining to that same person what a peptide fragmentation spectrum is.

The goal of this article is to lower that energy barrier by explaining in simple terms how a tandem mass spectrometry experiment works and what are the key research problems associated with this type of data.

## A Typical Shotgun Proteomics Experiment

**Figure 1:**
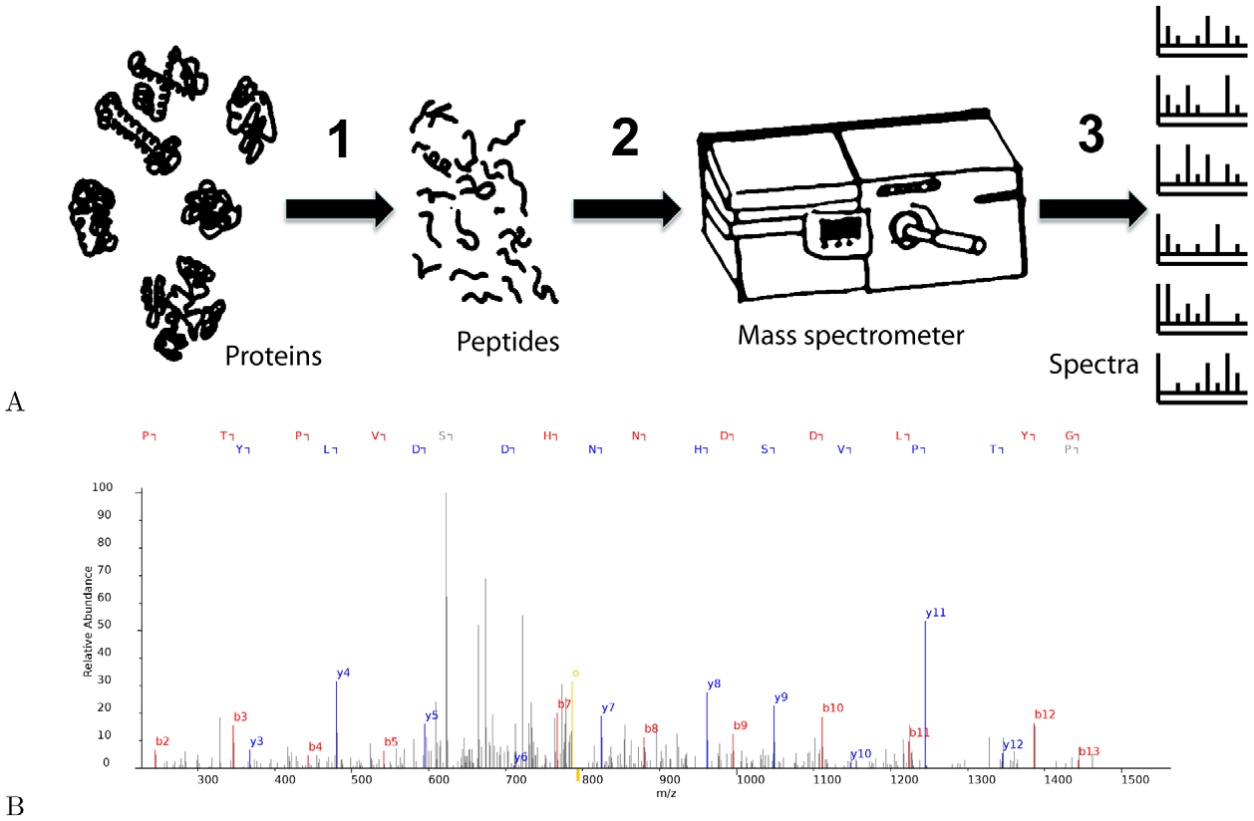
Overview of shotgun proteomics data production. (A) Schematic of a typical shotgun proteomics experiment. The three steps—(1) cleaving proteins into peptides, (2) separation of peptides using liquid chromatography, and (3) tandem mass spectrometry analysis—are described in the text. (B) A sample fragmentation spectrum, along with the peptide responsible for generating the spectrum.

A typical shotgun proteomics experiment proceeds in three steps, as illustrated in Figure 1A. The input to the experiment is a collection of proteins, which have been isolated from a complex mixture. A typical complex mixture may contain a few thousand proteins, ranging in abundance from tens of copies to hundreds of thousands of copies.

In the first experimental step, the proteins are digested into peptides using a protease. This digestion is necessary because whole proteins are too massive to be subject to direct mass spectometry analysis without using very expensive equipment. Second, the peptides are subjected to liquid chromatography, in which the peptides pass through a thin glass column that separates the peptides based on a particular chemical property (e.g., the hydrophobicity). This separation step reduces the complexity of the mixtures of peptides going into the mass spectrometer. The third step, which occurs inside the mass spectrometer, involves two rounds of mass spectrometry. Approximately every second, the device analyzes the population of 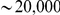 intact peptides that most recently exited from the liquid chromatography column. Then, based on this initial analysis, the machine selects approximately five distinct peptide species for fragmentation. Each of these fragmented species is isolated and subjected to a second round of mass spectrometry analysis. The resulting “fragmentation spectra” are the primary output of the experiment.

A sample fragmentation spectrum is shown in Figure 1B. During the fragmentation process, each amino acid sequence is typically cleaved once, so cleavage of the population results in a variety of observed prefix and suffix sequences. Each of these subpeptides is characterized by its mass-to-charge ratio (m/z, shown on the horizontal axis) and a corresponding intensity (unitless, shown on the vertical axis). The primary analysis challenge is to infer, for each observed fragmentation spectrum, the peptide sequence that was responsible for generating the spectrum.

## Peptide and Protein Identification

### The Spectrum Identification Problem

The spectrum identification problem is difficult to solve primarily because of noise in the observed spectrum. In general, the x-axis of the observed spectrum is known with relatively high precision. However, in any given spectrum, many expected fragment ions will fail to be observed, and the spectrum is also likely to contain a variety of additional, unexplained peaks. These unexplained peaks may result from unusual fragmentation events, in which small molecular groups are shed from the peptide during fragmentation, or from contaminating molecules (peptides or other small molecules) that are present in the mass spectrometer along with the target peptide species.

In practice, solutions to the spectrum identification problem fall into four general categories. By far the most commonly used approach is database search. The first computer program to use a database search procedure to identify fragmentation spectra was SEQUEST [3], and SEQUEST's basic algorithm (not including the function used to score individual peptide-spectrum matches) is still used by essentially all database search tools available today. The approach is as follows. We are given a spectrum 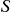, a peptide database 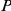, a precursor mass 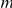 (i.e., the observed mass of the intact peptide), and a user-specified precursor mass tolerance 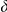. The algorithm extracts from the database all peptides whose mass lies within the range 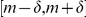. These comprise the set of *candidate peptides*

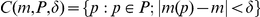
where 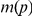 is the calculated mass of peptide 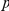. In practice, depending on the size of the peptide database and the precursor mass tolerance, the number of candidate peptides ranges from hundreds to hundreds of thousands. Each candidate peptide 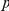 is compared to the observed spectrum using a score function 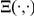. Frequently, the score function generates a theoretical spectrum for the given peptide and then compares the observed and theoretical spectra to one another. The program reports the candidate peptide that scores highest with respect to the observed spectrum:
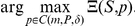
Database search methods differ primarily in their choice of score function.

An alternative to database search is de novo spectrum identification, in which the “database” of candidate peptides consists of the entire universe of possible amino acid sequences. A variety of graph-based dynamic programming methods can efficiently solve this problem, but in practice many spectra do not contain sufficient information to uniquely identify the correct peptide. Consequently, de novo identification methods generally fail to provide as many correct identifications as database search methods. Conversely, of course, de novo approaches are necessary when a peptide database is unavailable—i.e., for analysis of organims whose genomes have not yet been sequenced—or when the user is interested in identifying novel protein isoforms or polymporphisms.

Tag-based methods occupy an appealing middle ground between database search and de novo methods. Here, the basic idea is to use de novo analysis to identify a collection of subpeptides (“tags”) that are hypothesized to occur in the sequence, and then extract candidates from a database that contain the tags. Tag-based methods can be quite fast, and retain the ability to partially identify spectra for which the corresponding peptide is not in the database.

Finally, so-called library search methods identify spectra by comparing them to a library of previously identified spectra. These methods suffer a bit from a chicken-and-egg problem, in the sense that you must first somehow identify the spectra that go into the library. However, once you have successfully built such a library, searching an observed spectrum against real spectra is likely to give better results than searching against theoretical spectra. The caveat is that you have to be sure that your library does not contain false positives or chimeric spectra, i.e., spectra that were generated by a heterogeneous population of two or more co-eluting peptides.

Machine learning methods have been applied extensively to the spectrum identification problem, primarily as post-processors that discriminate between correct and incorrect identifications. Using methods such as support vector machines [4], linear discriminant analysis [5], or decision trees [6], these methods can dramatically increase the percentage of spectra from a given experiment that are confidently identified. Particularly powerful are semi-supervised learning methods [7], [8] that dynamically adjust their ranking scheme on the basis of characteristics of a given data set.

### Protein Identification

Once the peptide responsible for generating each observed spectrum has been identified, the downstream task of deciding which proteins are present in the sample seems like it should involve a straightforward process of aggregating evidence over all the spectra associated with a given protein. Unfortunately, this task is made much more difficult by the presence of so-called degenerate peptides, i.e., peptides that occur in multiple proteins.

Protein identification algorithms have improved significantly over the last decade. Early methods used simple heuristics to identify high-confidence proteins that contain a specified number of high-confidence peptide assignments [9]. A more sophisticated version of this approach employs a parsimony argument and attempts to find a minimal set of proteins that explain the observed identified spectra [10]. The most widely used method is pseudo-probabilistic, employing an expectation-maximization-like procedure to apportion evidence from each degenerate peptide among its corresponding parent proteins [11]. Other, related approaches either handle peptide degeneracy in a similar, heuristic fashion [12]–[14] or ignore the degeneracy entirely [15], [16]. Only recently have several groups proposed algorithms that directly solve the full protein identification problem within a rigorous probabilistic framework [17], [18].

### Computational and Statistical Challenges

Despite the almost dizzying array of existing methods for identifying peptides and proteins from shotgun proteomics data (reviewed in [19]), many significant analytical challenges remain. Perhaps most obvious is the need for algorithms that successfully identify proteins that are not in the database, either because they are polymorphic or because they contain post-translational modifications. The difficulty here is two-fold: how to make the search efficient, and how to successfully control the rate of false positive identifications, especially in the case when a wide variety of polymorphisms or modifications are allowed.

More fundamentally, the spectrum identification problem could likely benefit from the application of a rich, generative model of the peptide fragmentation process. Several such models have been described in the spectrum identification literature [20], [21], but none take into account the relatively rich literature on peptide fragmentation (reviewed in [22]).

A source of ongoing confusion and controversy in the field is the assignment of statistical confidence estimates to spectrum, peptide, or protein identifications. Varying protocols have been proposed, based upon empirical null distributions created by searching the spectra against a “decoy” database of shuffled or reversed peptide sequences [23], [24], upon procedures that involve fitting a parametric distribution to the empirical score distribution [25]–[27], or upon score functions for which exact *p*-values can be calculated under a simple zero-order Markov null model of peptides [28]. Critical assessment and comparison of many of these methods, especially with respect to potential biases incurred during evaluation of new algorithsm, is lacking in the literature. Furthermore, extending a statistic that is calculated with respect to individual spectra up to the peptide and protein levels is a non-trivial and relatively unexplored subject.

Historically, the development of tools for analyzing shotgun proteomics data has occurred in a stepwise fashion. The result is that small subtasks—assigning charge states to spectra, mapping peptides to spectra, re-ranking and assigning statistical confidence to spectrum identifications, and computing protein-level posteriors—have been solved separately. Clearly, in the long run, this piecemeal approach should be replaced by a joint model in which all relevant aspects of the experiment are taken into account. Such a joint model has the potential to model dependencies among variables at the spectrum, peptide, and protein level that are currently decoupled.

A perhaps equally challenging task is to convey the results of such a rich model to the user in a useful fashion. With current probabilistic protein identification tools, the standard approach of reporting a ranked list of proteins is insufficient. Even when we allow the list to contain groups of redundant proteins (i.e., protein isoforms that are indistinguishable on the basis of the observed spectra), crucial information about dependencies among the protein identifications is lost. For example, it is difficult to convey the information that, e.g., either protein A or protein B was present in the sample, but probably not both.

Another area where proteomics is in great need of guidance is experimental design. Proteomics experiments can be expensive and time consuming, yet most are being pursued without proper care in minimizing batch or systematic effects. Furthermore, most proteomics experiments are not powered correctly. Like gene expression analyses previouly, proteomics practices could benefit greatly from lessons learned in experimental design from classical statistics.

## Protein Quantification

The next logical step, after developing methods to *identify* the proteins in a complex mixture, is to develop methods to *quantify* the proteins. Quantification provides a more complete picture of the molecular contents of the cell, and allows us to generate or test specific hypotheses regarding the relationship between protein abundance and fundamental cellular processes or disease states.

Existing methods for protein quantification fall into three categories. *Stable isotope labeling* methods [29] perform relative protein quantification by incorporating a distinct heavy isotope tag to a sample to use as an internal standard. This labeled internal standard is then mixed into one or more other samples, and the relative signal intensity of the peptide measured by the mass spectrometer is compared to the measured intensity of the same peptide containing the heavy stable isotope label. This type of approach is quite powerful but the isotopic labeling step imposes significant overhead and limits the general applicability of these methods. *Spectral counting* methods [30]–[33] rely on counting the number of spectra that map to a given protein across multiple experiments. Spectral counting methods are not very accurate, because these methods fail to take into account the data-dependent acquisition that leads to the selection of peptides for fragmentation. However, spectral counting is appealing because the required counts are relatively easy to compute. Finally, *peptide chromatographic peak intensity* methods [34]–[37] use the area under the precursor ion peak as a proxy for peptide abundance. In contrast to spectral counting, methods based on peak areas are potentially much more accurate, but these methods require highly reproducible liquid chromatography as well as accurate methods for chromatographic alignment and identification of relevant spectral features.

Most of the mass spectrometry quantification literature focuses on measuring the relative abundance of the same protein across different samples. This allows, for example, comparing protein expression across different patients, tissues, or developmental stages. More difficult is estimating the relative abundance of two proteins within the same sample. For this task, the most commonly used, low-throughput approach is to calibrate the response of an individual peptide in a targeted selective reaction monitoring method (described in the next section). For large-scale quantification of many proteins, a few methods use a standard shotgun proteomics experiment, and either use a simple learning procedure [30] or rely on the observation that the three most intense peptides ionize similarly between proteins [38], [39].

## Targeted Proteomics

Many high-throughput proteomics experiments aim to identify or quantify all proteins in a complex sample. In contrast, selected reaction monitoring (SRM) experiments [40], [41] seek to quantify a smaller, specified set of proteins, e.g., a panel of biomarkers or members of a pathway of interest.

In an SRM experiment, the mass spectrometer is set to monitor the m/z values for a small number of peptides, as well as a specific fragment ion for each peptide. Each m/z pair, corresponding to the intact peptide and its fragment ion, is called a *transition*. Monitoring a small number of transitions, rather than scanning the entire m/z range, yields dramatically increased sensitivity relative to conventional “full scan” techniques. The goal of SRM is to select transitions that best detect the proteins of interest, subject to the constraint that such experiments can monitor only 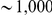 transitions per run in an automated fashion [42]. Thus, two complementary criteria must be optimized: (1) detect as many proteins of interest as possible and (2) accurately estimate the abundance of the monitored proteins.

Existing SRM pipelines typically focus on so-called proteotypic peptides [43] that can be easily observed in a mass spectometry experiment and that uniquely identify a specific protein. A variety of methods exist for identifying proteotypic peptides, either based on empirical rules [44], [45] or machine learning methods [43], [46], [47]. After identifying these peptides, SRM protocols typically use them as independent protein identifiers [48], with each peptide contributing equally to the evidence for that protein. What is missing is a method to search for a panel of peptides that jointly provide high quality quantification information about all of the target proteins.

## Outlook

Traditionally, most proteomics analysis has been carried out using relatively inexpensive ion trap instruments, which offer fairly low precision and accuracy on the m/z axis. Higher resolution instruments, which achieve precision of <10 ppm, were expensive and hence more rare. However, with the introduction of *Orbitrap* and improved quadrupole time-of-flight mass spectrometers [49], high-resolution instruments have become much more commonplace. Corresponding analytical methods that fully exploit the information available from high-resolution spectra have not used these data to their full potential.

In a mass spectrometer, intact peptides are characterized in an initial scan, followed by a series of secondary scans that characterize fragmented versions of the same peptides. The most common means of fragmenting peptides between the two scans is collision-induced dissociation, in which the charged peptides collide with neutral molecules such as helium, nitrogen, or argon. However, a variety of other fragmentation methods have been developed, including electron-capture dissociation, electron-transfer dissociation, and infrared multiphoton dissociation [50], and these can provide fragmentation spectra with quite different properties. More recently, some protocols have been developed to alternate between different types of fragmentation methods, with the aim of observing two complementary spectra representing each peptide [51]. Most existing analysis pipelines are tuned to handle one or two of the resulting types of spectra. Although some search engines allow users to select which types of fragmentation ions are included in the search, and some progress has been made recently toward developing score functions that can be adapted to various types of spectra [52], the field is still missing a generic analysis platform that can be adapted automatically and in a principled fashion to handle spectra produced by any given fragmentation protocol.

An interesting consideration is that as the scan speeds of tandem mass spectrometers increase, the difference between targeted and discovery proteomics will become more and more blurred. As an instrument becomes capable of collecting MS/MS spectra continuously across the chromatographic time-scale on an increasingly larger number of peptide precursors, the “m/z space” that remains unsampled will become less significant. Approaches that improve sampling by multiplexing the collection of fragmentation data are promising, but require methods to deconvolve the resulting mixed spectra. Such approaches fall under a general category known as *data-independent acquisition* because they collect their fragmentation data independently of whether a signal is observed within a particular precursor m/z window. Some initial attempts to interpret these data have shoe-horned traditional proteomics analysis pipelines to handle these data [53], [54]. Other workflows that have been developed specifically for these unique data have remained proprietary and, thus, not attracted much effort from the academic community to build and improve on these analyses.

One challenge that remains is enabling computational and experimental scientists pursuing proteomics to interact effectively. Frequently, experimentalists design new experiments using suboptimal analysis tools because they do not have the skills or knowledge to pursue alternatives. Likewise, computational scientists can expend a large amount of energy developing solutions to problems that are not interesting to experimentalists. The challenge remains how to get scientists from different disciplines that are based on different cultures and who speak different scientific languages to communicate and collaborate effectively. The RECOMB Satellite Conference on Computational Proteomics is one attempt to solve this problem. Speakers are brought from both the computational and experimental backgrounds. This meeting provides a forum for experimentalists to present problems that they are facing and for computational scientists to present algorithmic and statistical approaches that they have been developing. Ultimately, we need more meetings that foster collaboration between disciplines.

**Authors' Biographies**

**William Stafford Noble** (formerly William Noble Grundy) received the PhD in computer science and cognitive science from University of California (UC) San Diego in 1998. After a one-year postdoc with David Haussler at UC Santa Cruz, he became an Assistant Professor in the Department of Computer Science at Columbia University. In 2002, he joined the faculty of the Department of Genome Sciences at the University of Washington. He is now a Professor of Genome Sciences, with adjunct appointments in the Department of Computer Science and Engineering and the Department of Medicine. His research group develops and applies statistical and machine learning techniques for modeling and understanding biological processes at the molecular level. Dr. Noble is a member of three editorial boards, is the recipient of National Science Foundation CAREER award, and is a Sloan Research Fellow.

**Michael J. MacCoss** has been working with mass spectrometry instrumentation since 1994 when he was an undergraduate in a stable isotope geochemistry lab at the University of Vermont. He became interested in biomedical applications working in Dr. Patrick Griffin's protein mass spectrometry lab at Merck Research Laboratories during two summer internships in 1995 and 1996. In 2001, he completed a PhD in analytical chemistry with Professor Dwight Matthews in the development of stable isotope and mass spectrometry methodologies for the measurement of human amino acid and protein metabolism. After completing his degree, Dr. MacCoss moved to The Scripps Research Institute to work with Professor John R. Yates III as a postdoctoral fellow. Dr. MacCoss moved to the University of Washington in 2004 as an Assistant Professor of Genome Sciences, where his lab has focused on the development and application of mass spectrometry–based technologies for the high-throughput characterization of complex protein mixtures. In 2009 he was promoted to Associate Professor.

## References

[1] Liepe J, Mishto M, Textoris-Taube K, Janke K, Keller C (2010). The 20s proteasome splicing activity discovered by splicemet.. PLoS Comput Biol.

[2] Marchese R, Grandori R, Carloni P, Raugei S (2010). On the zwitterionic nature of gas-phase peptides and protein ions.. PLoS Comput Biol.

[3] Eng JK, McCormack AL, Yates JR (1994). An approach to correlate tandem mass spectral data of peptides with amino acid sequences in a protein database.. J Am Mass Spectrom.

[4] Anderson DC, Li W, Payan DG, Noble WS (2003). A new algorithm for the evaluation of shotgun peptide sequencing in proteomics: support vector machine classification of peptide MS/MS spectra and sequest scores.. J Proteome Res.

[5] Keller A, Nesvizhskii AI, Kolker E, Aebersold R (2002). Empirical statistical model to estimate the accuracy of peptide identification made by MS/MS and database search.. Anal Chem.

[6] Elias JE, Gibbons FD, King OD, Roth FP, Gygi SP (2004). Intensity-based protein identification by machine learning from a library of tandem mass spectra.. Nat Biotechnol.

[7] Käll L, Canterbury J, Weston J, Noble WS, MacCoss MJ (2007). A semi-supervised machine learning technique for peptide identification from shotgun proteomics datasets.. Nat Methods.

[8] Choi H, Nesvizhskii AI (2008). Semisupervised model-based validation of peptide identifications in mass spectrometry-based proteomics.. J Proteome Res.

[9] Tabb DL, McDonald WH, Yates JR (2002). DTASelect and Contrast: tools for assembling and comparing protein identifications from shotgun proteomics.. J Proteome Res.

[10] Ma ZQ, Dasari S, Chambers MC, Litton M, Sobecki SM (2009). IDPicker 2.0: Improved protein assembly with high discrimination peptide identification filtering.. J Proteome Res.

[11] Nesvizhskii AI, Keller A, Kolker E, Aebersold R (2003). A statistical model for identifying proteins by tandem mass spectrometry.. Anal Chem.

[12] Searle BC (2010). Scaffold: A bioinformatic tool for validating ms/ms-based proteomic studies.. Proteomics.

[13] Price TS, Lucitt MB, Wu W, Austin DJ, Pizarro A (2007). EBP, a program for protein identification using multiple tandem mass spectrometry datasets.. Mol Cell Proteomics.

[14] Feng J, Naiman DQ, Cooper B (2007). Probability-based pattern recognition and statistical framework for randomization: modeling tandem mass spectrum/peptide sequence false match frequencies.. Bioinformatics.

[15] Shen C, Wang Z, Shankar G, Zhang X, Li L (2008). A hierarchical statistical model to assess the confidence of peptides and proteins inferred from tandem mass spectrometry.. Bioinformatics.

[16] Li Q, MacCoss MJ, Stephens M (2010). A nested mixture model for protein identification using mass spectrometry.. Annals of Applied Sciences.

[17] Li YF, Arnold RJ, Li Y, Radivojac P, Sheng Q, Vingron M, Wong L (2008). A Bayesian approach to protein inference problem in shotgun proteomics.. Proceedings of the Twelfth Annual International Conference on Computational Molecular Biology.

[18] Serang O, MacCoss MJ, Noble WS (2010). Efficient marginalization to compute protein posterior probabilities from shotgun mass spectrometry data.. J Proteome Res.

[19] Nezvizhskii AI (2010). A survey of computational methods and error rate estimation procedures for peptide and protein identification in shotgun proteomics.. Journal of Proteomics.

[20] Wan Y, Chen T (2005). PepHMM: A hidden Markov model based scoring function for mass spectrometry database search.. Anal Chem.

[21] Klammer AA, Reynolds SR, Hoopmann M, MacCoss MJ, Bilmes J (2008). Modeling peptide fragmentation with dynamic Bayesian networks yields improved tandem mass spectrum identification.. Bioinformatics.

[22] Barton SJ, Whittaker JC (2009). Review of factors that inflence the abundance of ions produced in a tandem mass spectrometer and statistical methods for discovering these factors.. Mass Spectrom Rev.

[23] Käll L, Storey JD, MacCoss MJ, Noble WS (2008). Assigning significance to peptides identified by tandem mass spectrometry using decoy databases.. J Proteome Res.

[24] Elias JE, Gygi SP (2007). Target-decoy search strategy for increased confidence in large-scale protein identifications by mass spectrometry.. Nat Methods.

[25] Fenyo D, Beavis RC (2003). A method for assessing the statistical significance of mass spectrometrybased protein identification using general scoring schemes.. Anal Chem.

[26] Klammer AA, Park CY, Noble WS (2009). Statistical calibration of the sequest XCorr function.. J Proteome Res.

[27] Spirin V, Shpunt A, Seebacher J, Gentzel M, Shevchenko A (2011). Assigning spectrum-specific p-values to protein identifications by mass spectrometry.. Bioinformatics.

[28] Kim S, Gupta N, Pevzner PA (2008). Spectral probabilities and generating functions of tandem mass spectra: a strike against decoy databases.. J Proteome Res.

[29] Gevaert K, Impens F, Ghesquière B, Van Damme P, Lambrechts A (2008). Stable isotopic labeling in proteomics.. Proteomics.

[30] Lu P, Vogel C, Wang R, Yao X, Marcotte EM (2006). Absolute protein expression profiling estimates the relative contributions of transcriptional and translational regulation.. Nat Biotechnol.

[31] Griffin NM, Yu J, Long F, Oh P, Shore S (2010). Label-free, normalized quantification of complex mass spectrometry data for proteomic analysis.. Nat Biotechnol.

[32] Paoletti AC, Parmely TJ, Tomomori-Sato C, Sato S, Zhu D (2006). Quantitative proteomic analysis of distinct mammalian mediator complexes using normalized spectral abundance factors.. Proc Natl Acad Sci U S A.

[33] Old WM, Meyer-Arendt K, Aveline-Wolf L, Pierce KG, Mendoza A (2005). Comparison of label-free methods for quantifying human proteins by shotgun proteomics.. Mol Cell Proteomics.

[34] Clough T, Key M, Ott I, Ragg S, Schadow G (2009). Protein quantification in label-free LC-MS experiments.. J Proteome Res.

[35] Karpievitch Y, Stanley J, Taverner T, Huang J, Adkins JN (2009). A statistical framework for protein quantitation in bottom-up MS-based proteomics.. Bioinformatics.

[36] Bondarenko PV, Chelius D, Shaler TA (2002). Identification and relative quantitation of protein mixtures by enzymatic digestion followed by capillary reversed-phase liquid chromatography-tandem mass spectrometry.. Anal Chem.

[37] Roy SM, Becker CH (2007). Quantification of proteins and metabolites by mass spectrometry without isotopic labeling.. Methods Mol Biol.

[38] Silva JC, Gorenstein MV, Li GZ, Vissers JP, Geromanos SJ (2006). Absolute quantification of proteins by LCMSE: a virtue of parallel MS acquisition.. Mol Cell Proteomics.

[39] Malmstrom J, Beck M, Schmidt A, Lange V, Deutsch EW (2009). Proteome-wide cellular protein concentrations of the human pathogen *Leptospira interrogans*.. Nature.

[40] Kuhn E, Wu J, Karl J, Liao H, Zolg W (2004). Quantification of C-reactive protein in the serum of patients with rheumatoid arthritis using multiple reaction monitoring mass spectrometry and 13C-labeled peptide standards.. Proteomics.

[41] Lange V, Picotti P, Domon B, Aebersold RH (2008). Selected reaction monitoring for quantitative proteomics: a tutorial.. Mol Syst Biol.

[42] Lange V, Malmstrom JA, Didion J, King NL, Johansson BP (2008). Targeted quantitative analysis of *streptococcus pyogenes* virulence factors by multiple reaction monitoring.. Mol Cell Proteomics.

[43] Mallick P, Schirle M, Chen SS, Flory MR, Lee H (2006). Computational prediction of proteotypic peptides for quantitative proteomics.. Nat Biotechnol.

[44] Bronstrup M (2004). Absolute quantification strategies in proteomics based on mass spectrometry.. Expert Reviews of Proteomics.

[45] Kirkpatrick DS, Gerber SA, Gygi SP (2005). The absolute quantification strategy: a general procedure for the quantification of proteins and post-translational modifications.. Methods.

[46] Tang H, Arnold RJ, Alves P, Xun Z, Clemmer DE (2006). A computational approach toward label-free protein quantification using predicted peptide detectability.. Bioinformatics.

[47] Webb-Robertson BJM, Cannon WR, Oehmen CS, Shah AR, G V (2008). A support vector machine model for the prediction of proteotypic peptides for accurate mass and time proteomics.. Bioinformatics.

[48] Kuster B, Schirle M, Mallick P, Aebersold RH (2005). Scoring proteomes with proteotypic peptide probes.. Nat Rev Mol Cell Biol.

[49] Olsen JV, de Godoy LMF, Li G, Macek B, Mortensen P (2005). Parts per million mass accuracy on an orbitrap mass spectrometer via lock mass injection into a C-trap.. Mol Cell Proteomics.

[50] Sleno L, Volmer DA (2004). Ion activation methods for tandem mass spectrometry.. J Mass Spectrom.

[51] Swaney DL, McAlister GC, Coon JC (2008). Decision tree-driven tandem mass spectrometry for shotgun proteomics.. Nat Methods.

[52] Kim S, Mischerikow N, Bandeira N, Navarro JD, Wich L (2010). The generating function of CID, ETD, and CID/ETD pairs of tandem mass spectra: applications to database search.. Mol Cell Proteomics.

[53] Venable JD, Dong MQ, Wohlsclegel J, Dillin A, Yates JR (2004). Automated approach for quantitative analysis of complex peptide mixtures from tandem mass spectra.. Nat Methods.

[54] Panchaud A, Jung S, Shaffer SA, Aitchison JD, Goodlett DR (2011). Faster, quantitative, and accurate precursor acquisition independent from ion count.. Anal Chem.

